# Mathematical models for translational and clinical oncology

**DOI:** 10.1186/2043-9113-3-23

**Published:** 2013-11-07

**Authors:** Ralf Gallasch, Mirjana Efremova, Pornpimol Charoentong, Hubert Hackl, Zlatko Trajanoski

**Affiliations:** 1 Biocenter, Division of Bioinformatics, Innsbruck Medical University, Innrain 80, 6020 Innsbruck, Austria

## Abstract

In the context of translational and clinical oncology, mathematical models can provide novel insights into tumor-related processes and can support clinical oncologists in the design of the treatment regime, dosage, schedule, toxicity and drug-sensitivity. In this review we present an overview of mathematical models in this field beginning with carcinogenesis and proceeding to the different cancer treatments. By doing so we intended to highlight recent developments and emphasize the power of such theoretical work.

We first highlight mathematical models for translational oncology comprising epidemiologic and statistical models, mechanistic models for carcinogenesis and tumor growth, as well as evolutionary dynamics models which can help to describe and overcome a major problem in the clinic: therapy resistance. Next we review models for clinical oncology with a special emphasis on therapy including chemotherapy, targeted therapy, radiotherapy, immunotherapy and interaction of cancer cells with the immune system.

As evident from the published studies, mathematical modeling and computational simulation provided valuable insights into the molecular mechanisms of cancer, and can help to improve diagnosis and prognosis of the disease, and pinpoint novel therapeutic targets.

## Introduction

Cancer is still one of the leading causes of death in the world and major efforts have been undertaken to improve diagnosis and therapy of common cancer types. Recently developed technologies (i.e. next generation sequencing) give us unprecedented opportunities to study individual cancer samples at the molecular level and to identify genomic variants and rearrangements [1]. This information will build the basis for the stratification of patients, and for personalized or precision medicine. The increasing complexity of the generated data utilizing various high-throughput technologies for characterizing the genome, epigenome, transcriptome, proteome, metabolome, and interactome pose considerable challenges and therefore plethora of bioinformatics methods and tools for the analysis have been developed [2]. However, the real value of the disparate datasets can be truly exploited only if the data is integrated and will then enable one to comprehensively study molecular mechanisms of cancer cells.

One possibility for data integration is the use of mathematical models. Modeling has been successfully applied in physiology for many decades but only recently the quality and the quantity of biomolecular data became available for the development of causative and predictive models. Due to their importance in cancer mathematical models have also been in the focus of theoretical investigators. For example application of theoretical techniques and the postulation of the "two hit" hypothesis in the early 70s led to the identification of tumor-suppressor genes [3]. Later, in a landmark paper it was shown that cancer results from evolutionary processes occurring within the body [4].

In the context of translational (i.e. from bench to bedside, or in other words: transforming scientific discoveries arising from laboratory to clinical applications) and clinical oncology, mathematical models can provide novel insights into tumor growth and progression, into tumor-related processes such as angiogenesis, the immune response, and the interaction with the tumor microenvironment, and into the development of drug resistance. Furthermore, modeling can support the clinical oncologists in the design of the treatment regime, dosage, schedule, toxicity and drug-sensitivity. Common treatments against the different types of cancers include surgery, radiation therapy, chemotherapy, targeted therapy or combinations of those to limit the progression of malignant disease, eradicate tumor cells and prolong survival. The information gained from mathematical models can also help in the development and efficacy of clinical trials and treatment protocols, and can accelerate the progress of clinical research in fighting cancer.

To the best of our knowledge there is currently no review study on mathematical models focusing on translational and clinical oncology applications except for a similar attempt made by Swierniak et al. [5] few years ago. We therefore initiated this work to provide an overview of the field and stimulate the discussion and the development of novel models. While mechanistic models have proven extremely valuable and provided novel insights, there are not considered here and we refer the readers to recent reviews [6-8]. Given the wealth of published studies using mathematical models in cancer, we by no means intended to provide a comprehensive picture. Rather, we selected several topics we believe are of relevance for the readers. Wherever possible, we refer to additional reviews in order to guide interested researchers.

We first highlight mathematical models for translational oncology comprising epidemiologic and statistical models, mechanistic models for carcinogenesis and tumor growth, as well as evolutionary dynamics models [9], which can help to describe and overcome a major problem in the clinic: therapy resistance. Next we review models for clinical oncology. It should be noted that a survey of application of modeling results in clinics was beyond the scope of this review. Rather, we provide an overview of the models with a special emphasis on therapy including chemotherapy, targeted therapy, radiotherapy, immunotherapy and interaction of cancer cells with the immune system. Table 1 shows the specific categories and the publications used in this work.

**Table 1:** Categories and mathematical models in translational and clinical oncology reviewed in this paper

**Translational oncology**	**Clinical oncology**
Biological processes	Treatment options
Carcinogenesis	Chemotherapy
[3,10-17]	[61-70]
Tumor-growth	Targeted therapy
[18-27]	[58,72,73,75-78]
Clonal evolution	Radiotherapy
[37,38,41-46]	[79-81]
Therapy resistance	Tumor immune-cell interaction/immunotherapy
[47-59]	[82-88]

### Mathematical models for translational oncology

#### Carcinogenesis and tumor-growth models

Early models that aimed to explain the dynamics of cancer progression were based on experimental and epidemiological data, which indicated that the cancer incidence is often rapidly increased with age and simple patterns could be observed at the population level. Fisher and Hollomon [10] presented a multicellular model in which mutations occur in different cells within the same cell population and only the combination of all mutations leads to cancer development. As an alternative to this theory, Nordling [11] suggested that mutations must occur sequentially in the same cell for transformation into cancer cell.

Most mathematical models of cancer progression descend from Armitage and Doll’s [12] multistage theory, which include major concepts for how to think about incidence, carcinogenesis, and progression. The theory states that carcinogenesis progresses through series of genomic alterations in a single cell and the age-specific incidence of cancers is predicted to increase with a power of age that is one less than the number of alterations. Two other studies, using data comparing inherited and noninherited cases in colon cancer [13] and retinoblastoma [3], provided additional empirical evidence for the multistage theory. Knudson used a statistical analysis of the incidence of retinoblastoma in children to explain the role of tumor suppressor genes in sporadic and inherited cancers. This work was later extended to a two-stage stochastic model for the process of cancer initiation and progression [14], which lead to important subsequent work [15,16] that helped with characterization of other suppressor genes such as APC in colon cancer and TP53, which is mutated in several human tumors.

Even though these models provided accurate descriptions of cancer incidence data, they were unable to relate the data with the functional changes associated with tumor progression. Since then the understanding of the molecular mechanisms underlying tumor initiation and progression has improved [17] and mechanistic models that use biological knowledge and biophysical laws to quantify and predict cancer progression were developed.

The growth and development of solid tumors occurs in two stages – avascular and vascular. The early spatio-temporal models [18,19] of avascular tumor growth describe the interactions between tumor cell population and nutrients and calculate the nutrient concentrations as a function of tumor spheroid radius that is changing due to the rate of cell proliferation. Significant progress was made with the development of new models [20,21] that introduced the interrelated concepts of cell movement and pressure.

Since tumor induced angiogenesis i.e. the growth of a network of blood vessels, is a crucial component of solid tumor growth, the basic models have been expanded to account for tumor growth during angiogenesis and the increase of tumor availability associated with the expanding vasculature. In order to make a transition from avascular to vascular growth, tumors may secrete diffusible substances called tumor angiogenic factors (TAF). The earliest continuum models of tumor angiogenesis [22] describe the growth of a capillary network in terms of capillary tip densities and capillary sprout densities in response to TAF. The mathematical models in angiogenesis have mostly focused on describing endothelial cell migration and proliferation through the extracellular matrix [23-27]. A comprehensive overview of models in this area can be found in [28]. Mathematical modeling of blood flow in tumor-induced capillary networks has been described in more recent studies [29-31].

The concept that the successful formation of a tumor depends on vascularization has resulted in developing cancer therapies designed to inhibit the tumor vasculature in order to deprive the tumor from oxygen and nutrients. Several models have focused on exploring the efficiency of such antiangiogenic treatments [32-34]. Using methods of optimal control theory to analyze drug dosing and treatment strategies these studies showed that the combination with other forms of therapy would be beneficial.

#### Clonal evolution models and therapy resistance

An important conceptual breakthrough in understanding cancer lies in Darwinian and ecological theories: cancer progression is an evolutionary process that results from accumulation of genetic and epigenetic variations in somatic cells [35,36]. Experimental evidences and recent advances in genetic sequencing technologies – that allowed identification of the genetic alterations in a cancer cell - have revealed the complexity and heterogeneity of cancer progression and have stimulated the use of evolutionary-based approaches in the study of cancer.

Several methods of population dynamics and evolutionary game theory were applied to account for the elementary principles of evolution that lead to tumor initiation and progression. In the earliest models, mutations accumulate in a population of constant or variable size, and they consider only one or two mutations [37,38]. Newer models are now being used to investigate how the sequence and timing of mutations and the environmental conditions influence tumor progression [39,40]. An in-depth review of models that describe the evolutionary dynamics of cancer can be found in [41].

Several studies have focused on the waiting time to cancer development, which may be defined as the time from the first presence of neoplasm, until a critical number of hits (driver mutations) are accumulated and initiate the growth of carcinoma. Beerenwinkel et al. [42] developed one of the first models that was based on genomic studies of colorectal cancer patients. They related the waiting time to the population size, mutation rate, and the advantage of the driver mutations and showed that selective advantage of mutations has the largest effect on the evolutionary dynamics of tumorigenesis. In a similar manner, Bozic et al. [43], by fitting their model to glioblastoma and pancreatic cancer data, estimated that driver mutations give an average fitness advantage of 0.4%.

Another characteristic of evolutionary processes is the influence of the local cellular environment on the tumor progression. The progress of tumor is characterized not only by the genetic and epigenetic changes accumulating in the cells, but also by the dynamic interactions between cells within the tumor and between the cells and the constantly changing microenvironment. The microenvironment provides a selective fitness landscape that includes competing for limited resources and active intracellular (initiation of cell proliferation and cell death) and extracellular control mechanisms (the immune system) that aim to restore homeostasis.

There are several studies that utilize mathematical modeling to predict and quantify the interactions of the tumor cells with the surrounding environment during tumor progression [44,45]. Gatenby et al. [46] developed a model of carcinogenesis according to which the tumor cells have to overcome six microenvironment barriers that appear as tumor cells proliferate. They proposed that the nature and sequence of the alterations during carcinogenesis are determined by the specific microenvironmental properties that prevent proliferation within changing adaptive landscapes.

An important clinical problem in cancer research that can be analyzed using modeling techniques is the development of resistance to targeted therapies. Resistance to drugs may develop as a consequence of genetic events such as point mutations or gene amplifications. The emergence of resistance to therapy as a result from a single mutational effect has been first introduced in a model of Coldman and Goldie [47]. More recent studies have also used point mutations to explain the evolutionary dynamics of drug resistant cancer cells [48-51]. Other models studied gene amplification as one of the mechanisms that has a strong influence on the evolution of drug resistance [52-56].

Foo et al. [57] designed a methodology that can be used to investigate optimal drug dosing schedules to avoid resistance conferred by one (epi)genetic mutation. In a recent study, Diaz et al. [58] showed that tumors became resistant to anti-EGFR antibodies as a result of emergence of resistance mutations in KRAS and other genes that were present in clonal subpopulation within the tumors before the initiation of the treatment. Analyzing data from 20 melanoma patients who received targeted therapy, Bozic et al. [59] found that simultaneous administration of two drugs is much more effective than sequential therapy. The improved understanding of the evolutionary dynamics of cancer provided by these models can have practical implications in the design and administration of new cancer therapeutics.

**Figure 1:**
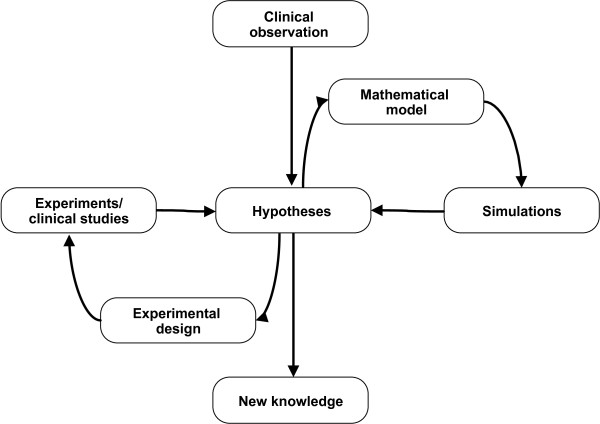
**Cycles of experiments and modeling for gaining new knowledge.** Experiments and clinical studies are closely coupled with mathematical modeling and simulations and require multiple cycles of iterations.

In summary, using the overwhelming amount of generated knowledge in tumor biology, mathematical modelers have succeeded in formalizing this knowledge and make it usable for simulations. Moreover, the published models represent a unique basis for testing novel hypotheses which are otherwise difficult or even impossible to test. For example, it is very difficult to obtain samples from early cancer stages or longitudinal samples in order to study the development of tumor heterogeneity. The models presented above enable researchers to address questions which were previously not possible and by using iterative cycles of simulations and experimentation ultimately lead to novel knowledge (Figure 1). Moreover, the maturity of the tools and the availability of data in public databases are additionally supporting the translation of this knowledge into clinical practice.

### Mathematical models for clinical oncology

#### Chemotherapy and targeted therapy

Chemotherapy is widely used therapy against cancer. Proliferating cells undergo different phases during the cell cycle including DNA replication and cell division and different chemotherapeutic compounds are affecting cells in different phases. The greatest challenge associated with chemotherapy is that not only cancer cells but also normal dividing cells are affected. In contrast, targeted cancer therapy [60] is aimed at specific molecular targets and is therefore more effective and has fewer side effects.

Fister et al. [61] developed a model that try to solve the problem of maximizing the effect on cancer cells but also maximizing the survival of the host cells. The mathematical model shows that if shorter periods of treatment are used it is possible to use higher doses of the drug and destroy more cancer cells without causing excess damage to the host cells. A more complex model is the cellular automaton model of Byrne et al. [62]. It is a multi-scale model that has a vascular layer, a cellular layer and an intracellular layer. With this model it is possible to get detailed spatio-temporal information about the tumor and the healthy tissue. In general three results are possible with the model; the tumor is eliminated, the tumor continues grow, or an oscillation. It is possible to combine different treatments in one model. The model of de Pillis et al. [63] based on a previous model [64] combines different treatments and shows that if the chemotherapy is stopped a system with a undetectable tumor can return to a high tumor state.

The combination of different treatments is one possibility to eliminate the patient’s tumor. Jackson et al. [65] introduced two different types of cells and investigated the tumors response to different chemotherapeutic strategies. It was possible to estimate the largest size of a tumor that can be eradicated by a bolus injection. With only one cell population the results of bolus and continues drug were similar. With two populations, one of them drug resistant, the continuous infusion increased the time to cure. This indicates that it is important to tailor treatment strategies.

Another interesting aspect is the use of growth factors in the model from Panetta and Adam [66]. They showed that the use of growth factors in their model increases the cell killing up to 20%. Based on the model of Anderson and Chaplain [27] McDougall [29] developed a model where the blood viscosity, pressure drop and mean capillary radii can be varied of the surrounding vessels of a solid tumor can be analyzed. The model shows that if there are highly interconnected vessels around the tumor there is a low drug delivery to the tumor itself. It shows that it is important to consider the vasculature around the tumor to find the optimal chemotherapy strategy.

The strategy of chemotherapy in combination with other treatments is being increasingly used. Powathil addressed this in recent publications [67-69] and showed ways to simulate and improve protocols of chemotherapy. It was demonstrated that the cytotoxic effect is dependent on many factors like timing of the drug delivery, time delay between the doses, heterogeneities of the cell cycle, the spatial distribution of the tumor and the surrounding microenvironment. It is noteworthy that these issues have been also investigated in older studies, e.g. using models of phase-specificity of chemotherapeutic drugs published in the 1990s [70]. In this section different methods were shown to model chemotherapy and effects that can lead to a better treatment strategy.

Most targeted cancer therapies use monoclonal antibodies directed against tumor-specific surface proteins or small-molecule drugs against intracellular targets (e.g. tyrosin kinases) [71]. Billy et al. [72] developed a model that simulates a treatment on the angiogenesis of tumors by gene therapy. The gene therapy is delivered by adenoviruses and influences, the antagonist of vascular endothelial growth factor, endostatin. The simulation showed that there is a critical treatment dose which is important to improve the efficacy.

TGF-β is a cytokine that has an immunosuppressive effect. In the model of Kirschner et al. [73] it was shown that a treatment with initial delivery of double stranded RNA into tumor cells that is cut by the enzyme Dicer into 21–23 segments known as siRNA inhibits TGF-β production and leads to a controlled oscillatory tumor behavior. Using a combination of experimental data and a mathematical model about the resistance against the monoclonal antibody panitumumab based on the Luria-Delbrück model [74], Diaz et al. [58] tested the development of mutations conferring resistance to the antibody. The simulation results suggested a combination of therapies where at least two pathways will be required. The use of ex-vivo activated alloreactive cytotoxic-T-lymphocytes (CTL) is another possibility to direct target the tumor. Kronik et al. [75] developed a mathematical model to investigate the effect of directly administrated CTL to glioblastomas. They showed that most sensitive parameters were the death rate of CTLs, the initial size of the tumor and the maximal growth rate.

Nanda et al. [76] developed a mathematical model simulating the drug imatinib mesylate that was approved in 2002 by the FDA for use in newly diagnosed cases of chronic myelogenous leukemia. The results show that a high dosing level from the beginning is optimal. Another interesting aspect of targeted therapy is the use of oncolytic viruses. Wein et al. [77] showed in their model that a single intratumoral injection in a solid tumor is not enough to effectively spread the virus. Also important is the suppression of the immune-mediated clearance of the virus. In the work of Mok et al. [78] two additional modifications are shown through mathematical modeling of herpes simplex viruses first the decreasing of the binding affinity of the virus and second the effective diffusion coefficient of the virus through degradation of the tumor extracellular matrix.

#### Radiotherapy

The aim of radiotherapy is to destroy the tumor cells but not the host cells. This is possible if the tumor cells are more sensitive to irradiation than the host cells. Mathematical modeling can show strategies and improve treatment protocols to obtain an optimal patient treatment. In this sense Rockne et al. [79] present a model to investigate the response to various schedules and dose distribution on a virtual tumor. The advantage in the mathematical simulation is that the effect of radiation can be observed continuously. The model suggests that a radiation dose on daily basis is more effective than several treatments per day.

Another important aspect is the general response of cells to radiation. Richard et al. [80] used a cellular automaton model to investigate these mechanisms after low doses of radiation. Enderling et al. [81] developed a model that simulates the recurrence after radiotherapy. In the 2D simulations it was shown that if pre-malignant cells reside in the breast post-surgery and survive radiotherapy this cells could be the reason for a recurrence.

#### Tumor immune-cell interaction and immunotherapy

The immune system plays an important role in tumor progression. Immune processes with different components like chemokines, cytokines or different cell types that work together are highly complex and intertwined. Mathematical modeling has already provided deeper insights and helped to get fundamental knowledge and improve patient’s treatment. For example De Boer et al. [82] developed a detailed model where they were able to show tumor regression and tumor growth dependent on the antigenicity of tumor-immune interaction. Tumour-infiltrating cytotoxic lymphocytes (TICLs) play an important role in tumor-immune interaction. Matzavinos et al. [83] developed a spatio-temporal model to investigate the interaction of TICLs and tumors. It is possible to simulate the spatio-temporal dynamics of TICLs in a solid tumor. Kirschner et al. [84] developed a model that includes immunotherapy with cultured immune cells that have anti-tumor reactivity and additionally IL-2. In simulations a total eradiation of the tumor was only possible with the immune therapy. In the model of de Pillis et al. [85] the cytolytic effectiveness of tumor specific T-cells was the most sensitive parameter. Following the simulation results the efficacy of the CD8+ T cells and the response to immunotherapy was correlating.

One therapy against superficial bladder cancer is the treatment with Bacillus Calmette-Guerin (BCG). Rentsch et al. [86] showed with their mathematical model that the dose of BCG and the treatment interval have a positive correlation of tumor extension. Wei [87] investigated this immunotherapy with a mathematical model and showed that the infection rate and the growth rate of the tumor are the most important parameters for a successful treatment. Rihan et al. [88] investigated the effect of adoptive cellular immunotherapy and found out that only a combination of the treatment with IL-2 can be used to clear the tumor.

In summary, major contributions for clinical oncology have been made by the modeling community. However, although many models were designed and tested for clinical applications, the use in routine setting is sparse. One way to overcome this is to develop models for very specific applications and rigorously test the performance and the predictive power. Furthermore, the use of the available knowledge should be also part of the decision process. We envision a computational decision support system which is using clinical data, molecular data, publicly available data, as well as simulation results of mathematical models to reach a decision for therapeutic strategy.

## Conclusion

In this review we presented an overview of mathematical models for translational oncology and clinical oncology beginning with carcinogenesis and proceeding to the different cancer treatments. By doing so we intended to highlight recent developments in the field and emphasize the power of this theoretical work. As demonstrated in a number of studies, mathematical modeling and computational simulation can provide valuable insights into the molecular mechanisms of cancer, can improve diagnosis and prognosis of the disease, and pinpoint novel therapeutic targets. As can be seen in Figure 1, it is often difficult to attribute the generation of new knowledge either to the modeling or to the experimental work. Regardless the origin, the insights obtained from such cycles of experiments and modeling can improve our understanding of the complexity of cancer progression and eventually be used to stop or at least slow down the processes of tumor initiation, evolution and resistance to therapies.

## Competing interests

The authors declare that they have no competing interests.

## Authors’ contribution

RG, ME, PC, HH, and ZT carried out literature search and wrote the manuscript. ZT conceived the study. All authors read and approved the final manuscript.
